# The role of PANSS symptoms and adverse events in explaining the effects of paliperidone on social functioning: a causal mediation analysis approach

**DOI:** 10.1038/s41537-018-0054-8

**Published:** 2018-06-27

**Authors:** Xue Zou, Yiwen Zhu, John W. Jackson, Andrea Bellavia, Garrett M. Fitzmaurice, Franca Centorrino, Linda Valeri

**Affiliations:** 1 Department of Biostatistics, Harvard T.H. Chan School of Public Health, Boston, MA USA; 2 Department of Epidemiology, Harvard T.H. Chan School of Public Health, Boston, MA USA; 3 Department of Epidemiology, Johns Hopkins Bloomberg School of Public Health, Baltimore, MD USA; 4 Department of Environmental Health, Harvard T.H. Chan School of Public Health, Boston, MA USA; 5 Department of Psychiatry, Harvard Medical School, Boston, MA USA; 6 Laboratory for Psychiatric Biostatistics, McLean Hospital, Belmont, MA USA

## Abstract

To date, no study has evaluated the joint role of symptoms and adverse events as mediators of the effect of second-generation antipsychotics on patients’ social functioning. We used recently developed methods for mediation analysis with multiple mediators to clarify the interplay of adverse events and symptoms in explaining the effects of paliperidone (R code for implementing the mediation analysis for multiple mediators is provided). We used data from 490 participants in a 6-week randomized dose–response trial that assigned three fixed dosages of ER OROS paliperidone (3, 9, and 15 mg/day). The primary outcome was an individual’s score on the social performance scale assessed after 6 weeks. The sum of Positive and Negative Syndrome Scale (PANSS), weight gain, and extrapyramidal symptoms measured via the Simpson–Angus Scale after 5 weeks were investigated as potential mediators and effect modifiers of treatment effects. Results from mediation analyses showed that the improvements in social functioning are partly explained by reduction in PANSS symptoms. Suggestive evidence that adverse events could play a role as mediators was found. In particular, weight gain displayed a non-linear relationship with social functioning, whereby beneficial effects observed at small levels of weight gain were reduced in the presence of excessive weight gain. In conclusion, we found that the short-term effects of paliperidone on social functioning were dependent on the successful reduction in PANSS symptoms and possibly the occurrence of excessive weight gain, thus suggesting future directions for treatment and interventions.

## Antipsychotic: weight gain “side effect” impacts drug benefit

People with schizophrenia who take the antipsychotic drug paliperidone show the greatest improvements in social functioning if their disease symptoms decrease and they experience small levels of weight gain. Linda Valeri from McLean Hospital in Belmont, Massachusetts, USA, and colleagues applied a statistical method called mediation analysis to study clinical data from a 490-person, 6-week trial involving an extended-release formulation of paliperidone. As expected, the researchers showed that an individual’s score on a social performance scale—the primary outcome in the trial—could be explained in part by reductions in both positive symptoms, such as hallucinations, and negative symptoms, such as apathy. But in addition, a “side effect” impacted social functioning in an inverted-U shaped manner, with modest increases in body weight proving beneficial but too many extra pounds having a detrimental effect.

## Introduction

The treatment goals for schizophrenia are to rapidly ameliorate or eliminate symptoms, prevent relapse, induce sustained recovery, and improve personal and social functioning. The Positive and Negative Syndrome Scale (PANSS) is the most popular scale used for measuring symptom severity in patients with schizophrenia. Positive symptoms refer to an excess or distortion of normal functions (e.g., hallucinations and delusions), and negative symptoms represent a diminution or loss of normal functions.^1^ Patient's personal and social functioning remains an area of deficit in patients with schizophrenia,^2^ with only limited data available regarding the effects of either atypical or conventional antipsychotic agents on this domain. Among patients affected by psychotic disorders, schizophrenia patients display the highest deficit in social functioning.^3,4^ Social functioning has been recognized as an important contributor to overall quality of life and a determinant of treatment success.^4^ Cognitive impairments impact functioning skills in schizophrenia patients and it is known that positive and negative symptoms add to the influence of cognitive impairments for prediction of real-world outcomes.^5,6^ A study assessing predictors of Social Skills Performance Assessment (SSPA)^7^ suggests that specific negative symptoms, including passive-apathetic social withdrawal, blunted affect, and lack of spontaneity are important predictors of SSPA, and particularly of the items capturing social outcomes. Positive symptoms of hallucinatory behavior and suspiciousness were found to predict amount of everyday activities.^8^ The Personal and Social Performance (PSP) scale has been developed to measure social functioning in schizophrenia.^9^ It includes four specific domains of assessment (socially useful activities including work and study; personal and social relationships; self-care; and disturbing and aggressive behaviors) and is a validated outcome measure in acute and stable patients with schizophrenia. Recent literature has shown that a substantial number of antipsychotic drug effects on PANSS occur during the first 2−4 weeks of treatment.^10^ It has been suggested that an initial symptom improvement and emergence of adverse events during the first week of treatment is a possible indicator of how well patients are going to respond later in treatment.^10^ The occurrence of side-effects such as extrapyramidal symptoms (EPS) and excessive weight gain (WG) may hamper the effects on their targeted outcomes, and social functioning in particular.^11^ As such, it is critical to evaluate the interplay of treatments and side-effects in a single framework, to clarify the role of these side-effects in explaining the observed efficacy of the treatments and potentially improving the care of patients. Paliperidone extended-release tablet (paliperidone ER) is an oral psychotropic agent developed for schizophrenia treatment. The efficacy and safety of once-daily paliperidone ER (3 mg, 9 mg, and 15 mg) and olanzapine were compared with placebo in 618 patients with acute schizophrenia in the Extended-Release ER OROS® paliperidone trial (trial registration number: NCT00083668), a 6-week, multicenter, double-blind, randomized, parallel group study. The trial demonstrated efficacy of the antipsychotic in improving both PANSS symptoms and social functioning.^12^ To the best of our knowledge, no studies have attempted to quantify the effect of paliperidone in terms of improved social performance score while simultaneously taking into account its effect on other secondary outcomes such as WG, PANSS, and EPS symptoms. These secondary outcomes may act both as modifiers, possibly antagonistically, of the treatment effects on patients’ social functioning (i.e., improvement may differ depending on the level of the symptoms and adverse events), and as mediators of the treatment effect (i.e., the secondary outcomes are involved in the mechanism through which the drug affects social functioning). The aim of this paper is to use recently developed methods in causal mediation analysis for multiple mediators^13,14^ to clarify and quantify the role of PANSS symptoms and adverse events in explaining the effect on social functioning of second-generation drugs for the treatment of schizophrenia. We address these questions using data from the Extended-Release ER OROS® paliperidone trial. Furthermore, we provide a discussion of the limitations and strengths of the adoption of these approaches in the context of clinical trials for schizophrenia.

## Methods

### Mediation analysis with multiple mediators

Mediation analysis allows decomposing a given treatment-outcome (or exposure-outcome) association (total effect) into the effect that operates through one or more intermediate variables of interest (indirect effects) and the effect that is due to other independent mechanisms (direct effect).^15,16^ Defining direct and indirect effects in counterfactual terms has been crucial for overcoming major limitations of the classical approaches to mediation, and the field of causal mediation analysis has rapidly expanded over the last decade.^17^

In the present study, we hypothesize that multiple mediators are simultaneously contributing to the treatment-outcome effect. In the context of our study, the treatment is paliperidone, the mediators are WG, PANSS, and EPS symptoms, and the outcome is social functioning. We are interested in estimating the direct effect of paliperidone on social functioning through pathways that are independent of all three mediators, and the indirect effects through each of the potential mediators. In particular, we seek to estimate the effect that paliperidone would have on social functioning if paliperidone could only change WG downstream, so that its effect was forced to be completely mediated through changes in PANSS. Similarly, we wish to estimate the indirect effects through EPS and WG as well. Estimating these effects becomes more challenging in the presence of treatment-mediator and mediator-mediator interactions.^13,14^ For a formal definition of the effects using the potential outcomes notation for causal inference the interested reader can refer to Bellavia and Valeri.^14^ To identify the direct and indirect effects, control must be made for a covariate set C that includes all confounders of not only the treatment-outcome relationship but also the mediator-outcome relationships. We formally require that there is no unmeasured confounding for the treatment -outcome relationship (Assumption 1), and no unmeasured confounding for the mediator-outcome relationship (Assumption 2). Furthermore, there must be no unmeasured confounding of the treatment-mediator relationships (Assumption 3). Finally, there must be no effect of treatment that itself affects both mediator and outcome, i.e., no mediator-outcome confounder that is itself affected by the treatment (Assumption 4).^18,19^ Assumptions 2, 3, and 4 are required to hold for all mediators included in the analysis. We describe the estimation strategy in the statistical analysis section.

### Study population

The Extended-Release ER OROS® paliperidone trial (NCT00083668) was a 6-week, multicenter, double-blind, randomized, placebo- and active-controlled, parallel group, dose–response study conducted between May 2004 and May 2005 at 74 centers (31 centers in North America and Canada, 17 in Eastern Europe, 12 in Asia, 5 in Israel, 5 in Mexico and 4 in South Africa) to assess the efficacy of ER OROS paliperidone compared with placebo in subjects with schizophrenia. Olanzapine was chosen as concurrent active control group to confirm that the study was adequate to detect a drug effect (i.e., assay sensitivity) in case the three ER OROS paliperidone treatment groups had failed to show efficacy. Participating patients were randomly assigned to receive placebo, or either 3, 9, and 15 mg/day doses of ER OROS paliperidone, or 10 mg/day of Olanzapine and followed-up for up to 6 weeks or until treatment was discontinued for any reason. The trial data are publicly available for secondary analyses through the Yale Open Data Access Project (http://yoda.yale.edu/). This study carries out an intent-to-treat analysis for treatment group comparisons. Further details on rationale, design, and methods have been described in previous publications.^12^ Inclusion criteria to the trial required participants to be 18–65 years of age and having received a diagnosis of schizophrenia, as determined on the basis of the Structured Clinical Interview of the Diagnostic and Statistical Manual of Mental Disorders, fourth edition, for at least 1 year. In total, 618 participants were included and 490 were considered for the current analyses, as the olanzapine arm was excluded from the current study.

An Institutional Review Board or an Independent Ethics Committee at each center approved the study protocol. All patients gave informed consent after the study procedure had been fully explained.

### Aims

We sought to: (a) investigate the main effects of the mediators, PANSS, WG, and EPS on social functioning as well as potential effects of treatment on the social functioning outcome modified by the hypothesized mediators; (b) quantify the importance of the pathways that involve PANSS, WG, and EPS; (c) investigate the direct effect of treatment through pathways independent of the adverse events and symptoms.

### Treatments

Patients were randomized into five groups: placebo, olanzapine (10 mg capsule), Paliperidone (Janssen Pharmaceuticals) (3 mg capsule), Paliperidone (Janssen Pharmaceuticals) (9 mg capsule), Paliperidone (Janssen Pharmaceuticals) (15 mg capsule). For additional details on doses and administration, procedures we refer to Davidson et al.^12^

### Outcome and mediators

The primary outcome of this study was patient functioning as determined by the PSP scale.^9^ The PSP scale is a 100-point single-item rating scale, with a score of 1–10 representing lack of autonomy in basic functioning, and 91–100 reflecting excellent functioning. The ratings are based mainly on the assessment of patient’s functioning in four main areas: (a) socially useful activities, including work and study; (b) personal and social relationships; (c) self-care; and (d) disturbing and aggressive behaviors. In the current study, we considered symptoms and adverse events as relevant secondary outcomes, which could act as moderators and/or mediators of the treatment effect on PSP. We considered positive symptoms (PANSS + ) and negative symptoms (PANSS-) scores assessed according to the standard criteria,^2^ with seven items to detect excess or distortion of normal functions (PANSS + ), and seven items to detect diminution or loss of normal functions (PANSS −). Each item was scored from 1 to 7, thus yielding a total score ranging from 7 to 49, for both PANSS + and PANSS −. In the analyses, we used the total PANSS sum of both positive and negative scores. For these scales, a higher score indicates more severe pathology. Further, we considered the metabolic adverse event of percent WG and neurological adverse events of extrapyramidal symptoms measured by the Simpson–Angus Rating Scale (SAS).^20^ PSP was collected at baseline and at the end of follow-up at week 6. PANSS symptoms and weight data were collected at baseline and every week of the study. Neurological adverse events were measured every week or at the time of occurrence of an event. For these analyses, we used the performance in PSP scores after 6 weeks at the end of follow-up, symptoms and percent WG after 5 weeks and the maximum of neurological adverse events scores post baseline. This was done to ensure that the secondary outcomes temporally preceded PSP scores (the primary outcome), which would be required to causally interpret results from a mediation analysis.

### Confounders

Potential baseline confounders evaluated in this study included: age (continuous, years); gender; race (categorical: white, black, others); relative day of disease onset (continuous); total PANSS score at baseline (continuous); baseline BMI (continuous); baseline PSP score (continuous); baseline Akathisia and SAS scores; study site.

Treatment randomization is expected to balance the treatment groups on most of the potential confounders. Nevertheless, when performing mediation analysis, randomization on the treatment does not assure that the same randomization will hold for the mediator (i.e., the association between the mediator and outcome will likely be confounded), even in expectation.^19^ For this reason, all statistical analyses we present are adjusted for the confounders listed above.

### Statistical analysis

All analyses in this study were performed by pairwise comparison of each paliperidone dose and placebo. To account for missing data in baseline covariates, outcome, and mediators, we employed multiple imputation techniques.^21^ Data were multiply imputed 50 times and analyses were conducted for each imputed data set. We combined the estimates of all analyses using Rubin’s rule.^21,22^

We first estimated multivariable-adjusted treatment effects on PSP scores, using linear regression. Multivariable-adjusted linear regression models were also used to estimate changes in percent WG, in the sum of PANSS + and PANSS− symptoms, and in SAS scores between Paliperidone doses and placebo.

We next investigated the secondary outcomes as possible effect modifiers or mediators of treatment effects on PSP scores. We tested for effect modification by including interaction terms between paliperidone treatment doses and each of the secondary outcomes in predicting PSP scores. By conducting a formal mediation analysis, we provided a valid assessment of post-treatment factors that could both mediate and modify the treatment-outcome association. The study sample size allowed for 80% power to detect mediated effects that explained > 25% of the total effect.

**Fig. 1:**
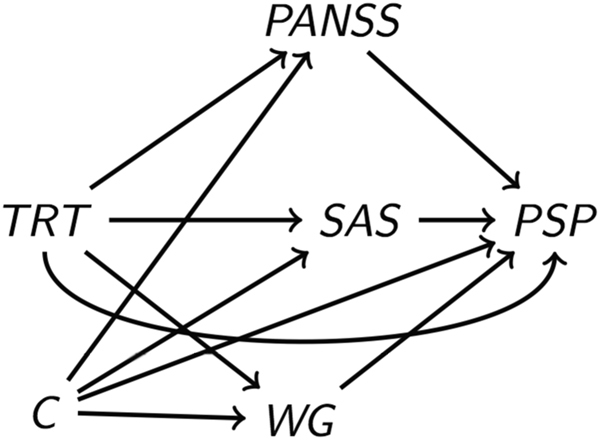
Direct acyclic graph representing the potential role of PANSS symptoms, weight gain (WG), and extrapyramidal symptoms measured via the Simpson–Angus Scale (SAS) as mediators of the effect of paliperidone on social functioning measured by PSP scores. Despite the randomization at the treatment level, this model requires taking into account potential confounders of the mediator-outcome association

We applied the parametric regression approaches for mediation analysis with multiple mediators^13,14^ to investigate the contribution of the secondary outcomes in the mechanism through which the antipsychotic treatment affects PSP score (Fig. 1). Estimators of the direct and indirect effects as functions of the coefficients of the outcome and mediators’ regressions were used to compute the effects and bootstrapping procedures were employed to obtain inferences. For a more in-depth description of the approach, the interested reader can refer to Bellavia and Valeri (2017).^14^

### Code availability

All analyses were performed with Rstudio (version 3.3.0), and all tests were two tailed. The R package ‘mice’ was used to conduct the multiple imputation procedure. R code of the mediation analysis with multiple mediators can be found in the Supplemental Files.

## Results

Table 1 presents the baseline characteristics of the study population by assigned treatment. The variable relative day of disease onset was missing for a third of the patients. Discontinuation rates appeared to be the highest in the paliperidone 3 mg group, with 16% missing information during the follow-up.

**Table 1:** Baseline characteristics of the study population by assigned treatment, in the whole sample (*n* = 490, excludes Olanzanzapine arm)

	Placebo *n*=123	Paliperidone 3 mg *n*= 27	Paliperidone 9 mg *n*=125	Paliperidone 15 g *n*=115	Missing^a^
Age, mean (sd, min, max)	37.5 (11.2, 18.0, 63.0)	36.2 (10.9, 19.0, 64.0)	36.0 (10.9, 18.0, 60.0)	37.8 (9.9, 18.0, 62.0)	0 (0)
Male, *n* (%)	86 (69.9)	81 (63.8)	81 (64.8)	74 (64.3)	0 (0)
Race					
Asian, *n* (%)	28 (22.8)	31 (24.4)	28 (22.4)	29 (25.2)	0 (0)
Black, *n* (%)	26 (21.1)	27 (21.3)	24(19.2)	28 (24.3)	0 (0)
Other, *n* (%)	6 (4.9)	7 (5.5)	8 (6.4)	7 (6.1)	0 (0)
White, *n* (%)	63 (51.2)	62 (48.8)	65 (52.0)	51 (44.3)	0 (0)
Country					
North American, *n* (%)	43 (35.0)	42 (33.1)	47 (37.6)	38 (33.0)	0 (0)
Asia/Pacific, *n* (%)	37 (30.1)	41 (33.1)	37 (29.6)	37 (32.2)	0 (0)
Europe and other, *n* (%)	33 (26.8)	34 (26.8)	32 (25.6)	31 (27.0)	0 (0)
Africa, *n* (%)	10 (8.1)	10 (7.9)	9 (7.2)	9 (7.8)	0 (0)
Systolic blood pressure, mean (sd, min, max)	120.1 (13.8, 89.0, 170.0)	118.2 (12.3, 90.0, 146.5)	119.6 (12.8, 90.0, 156.0)	120.3 (14.8, 95.0, 188.0)	5 (1.0)
Diastolic blood pressure, mean (sd, min, max)	75.8 (9.8, 54.0, 110.0)	74.9 (9.1, 50.5, 100.0)	77.0 (8.7, 55.5, 100.0)	75.9 (9.3, 59.0, 112.5)	5 (1.0)
BMI, mean (sd, min, max)	25.7 (5.6, 16.4, 48.1)	25.7 (5.7, 14.9, 44.6)	25.6 (5.9, 16.4, 55.5)	26.8 (7.7, 16.8, 57.3)	4 (0.8)
SAS score, mean (sd, min, max)	2.4 (4.0, 0.0, 20.0)	2.9 (5.0, 0.0, 23.0)	2.9 (4.7, 0.0, 30.0)	3.2 (5.6, 0.0, 27.0)	6 (1.2)
BARS score, mean (sd, min, max)	0.2 (0.5, 0.0, 2.0)	0.2 (0.6, 0.0, 3.0)	0.3 (0.7, 0.0, 4.0)	0.2 (0.5, 0.0, 2.0)	2 (0.4)
Total PANSS score, mean (sd, min, max)	47.5 (6.6, 33.0, 60.0)	46.4 (7.1, 32.0, 76.0)	48.2 (7.6, 31.0, 69.0)	47.0 (8.2, 0.0, 64.0)	0 (0)
PSS score, mean (sd, min, max)	50.0 (14.1, 20.0, 91.0)	48.5 (14.3, 17.0, 85.0)	49.6 (16.6, 20.0, 90.0)	47.6 (14.9, 20.0, 91.0)	2 (0.4)
pystdy, mean (sd, min, max)	−1672.3 (1632.6, −7626.0, 7)	−1694.2 (1873.2, −7349.0, −3)	−1716.5 (1757.6, −9749.0, −6)	−1970.2 (2085.2, −10412.0, −3)	145 (29.6)

### Outcome regressions: PSP scores

Table 2 (first column) presents the outcome regression output adjusted for treatment group and baseline covariates. At baseline, the average PSP score was similar across treatment arms and of about 48 points. Compared with placebo, patients assigned to paliperidone treatment groups experienced an increment in social functioning. The total effect of paliperidone on PSP scores was clinically relevant (paliperidone dose 3 mg: *β* = 8.42; 95% CI: 4.21, 12.64. Paliperidone dose 9 mg: *β* = 9.74; 95% CI: 6.12, 13.35. Paliperidone dose 15 mg: *β* = 13.03; 95% CI: 9.21, 16.84). Table 3 presents the outcome regression output further adjusting for the mediators. Once adjustment was made for the three mediators the treatment effects dramatically reduced (Paliperidone dose 3 mg: *β* = 3.34; 95% CI: − 1.29,7.98. Paliperidone dose 9 mg: *β* = 5.05; 95% CI: 1.32, 8.78. Paliperidone dose 15 mg: *β* = 6.61; 95% CI: 2.04, 11.18). We found evidence of a quadratic effect of WG, whereby a positive main effect of WG is coupled with a large and negative quadratic effect. For a WG above 19–33% we observed a negative effect on PSP for patients assigned to low to high doses of paliperidone. We found a strong association between PANSS scores and PSP. On the other hand, the association between SAS scores and the outcome was not statistically significant. There was neither evidence of treatment-mediator nor mediator-mediator interactions.

**Table 2:** Multivariable-adjusted differences between placebo and paliperidone in the primary outcome (PSP) and mediators (WG, PANSS, and SAS score)

	PSP	PANSS	WG	SAS
Paliperidone 3 mg	8.42 (4.21, 12.64)	−3.47(−6.47, −0.47)	0.02 (0.01, 0.03)	0.14 (−0.19, 0.48)
Paliperidone 9 mg	9.74 (6.12, 13.35)	−3.91(−6.77, −1.06)	0.03 (0.02, 0.05)	0.71 (0.27, 1.16)
Paliperidone 15 mg	13.03 (9.21, 16.84)	−4.78(−7.86, −1.71)	0.04 (0.02, 0.05)	0.68 (0.24, 1.13)
Placebo	Ref	Ref	Ref	Ref

All models adjusted for age, sex, race, country, relative day of disease onset, total PANSS score at baseline, PSP score at baseline, BARS score at baseline, SAS score at baseline, BMI at baseline. We report pooled estimates (pooled confidence intervals) from 50 multiple imputations

**Table 3:** Multivariable regression PSP after 6 weeks adjusting for treatment and the mediators PANSS score, WG, and SAS score

	Paliperidone 3 mg	Paliperidone 9 mg	Paliperidone 15 mg
Treatment	3.34 (−1.29, 7.98)	5.05 (1.32, 8.78)	6.61 (2.04, 11.18)
WG	73.28 (18.97, 127.59)	34.04 (−5.83, 73.91)	51.36 (11.16, 91.55)
WG_sq	−223.83 (−424.72, −22.94)	−177.01 (−347.44, −6.58)	−201.68 (−366.99, −36.37)
PANSS	−0.91(−1.16, −0.66)	−0.82 (−1.06, −0.58)	−0.81 (−1.05, −0.57)
Max SAS	0.92 (V0.66, 2.50)	0.31 (−0.69, 1.32)	0.80 (−0.21, 1.82)

All models adjusted for age, sex, race, country, relative day of disease onset, total PANSS score at baseline, PSP score at baseline, BARS score at baseline, SAS score at baseline, BMI at baseline. We report pooled estimates (pooled confidence intervals) from 50 multiple imputations

### Mediator regressions: percent WG, PANSS, and SAS

Table 2 (except the first column) presents the secondary outcomes regression outputs. All regression analyses were adjusted for baseline confounders.

### Percent WG

The average percent change in weight among patients in the placebo group was small (−1%). Compared with this reference group, the patients assigned to the paliperidone treatment groups displayed positive, albeit small, increments in weight. The percent WG calculated after 6 weeks from the beginning of the study for the paliperidone groups were respectively, 1% (sd = 4.0), 2% (sd = 4.0), and 3% (sd = 5.0). Significant effects were observed in comparison with placebo (Paliperidone dose 3 mg: *β* = 1.84%; 95% CI: 0.50, 3.14. Paliperidone dose 9 mg: *β* = 3.10; 95% CI: 1.66, 4.53. Paliperidone dose 15 mg: *β* = 3.86; 95% CI: 2.29, 5.44). An outlier of 42% increase in WG was noticed in placebo group. Sensitivity analysis were conducted excluding the outlier and showed no influence of this extreme value in the results.

### PANSS

At baseline the average PANSS score obtained by the sum of PANSS positive and negative scales was similar across treatment arms and of about 47 points. The sum of PANSS scores calculated after 5 weeks from the beginning of the study were 37.45 (sd = 10.6), 34.10 (sd = 8.9), 34.71 (sd = 8.2) and 35.02 (sd = 9.4). Lower PANSS scores were observed in patients assigned to paliperidone treatment groups whereby the higher dose, the stronger reduction effect (Paliperidone dose 3 mg: β = −3.47; 95% CI: −6.47, −0.47. Paliperidone dose 9 mg: β = −3.91; 95% CI: −6.77, −1.06. Paliperidone dose 15 mg: *β* = −4.78; 95% CI: −7.86,−1.71).

### SAS

The SAS scores, calculated as the maximum level experienced by patients assigned to low, medium, and high paliperidone doses between week 0 and week 5, were 2.8 (sd = 4.1), 3.6 (sd = 5.6), 4.0 (sd = 6.0), and 4.4 (sd = 6.5). Higher SAS score were observed in patients assigned to higher doses of treatment, where their maximum scores were 25.0, 33.0, 29.0, respectively. Significantly higher SAS scores relative to placebo were observed in patients assigned to 9 mg or 15 mg doses of paliperidone treatment (paliperidone dose 3 mg: *β* = 0.14; 95% CI: − 0.19, 0.48. Paliperidone dose 9 mg: *β* = 0.71; 95% CI: 0.26, 1.16. Paliperidone dose 15 mg: *β* = 0.68; 95% CI: 0.24, 1.13).

### Mediation analysis with multiple mediators

Table 4 presents the mediation analyses results. For the mediation analyses we did not consider SAS as a mediator, as the association between SAS and PSP score was not statistically significant (Table 3). Direct and indirect effects summed up to the total effect previously estimated. The direct effect of paliperidone treatment’s effect on PSP score through pathways independent of WG and PANSS is positive (Paliperidone dose 3 mg: DE = 3.53; 95% CI: −0.40, 7.46. Paliperidone dose 9 mg: DE = 5.16; 95% CI: 1.33, 8.99. Paliperidone dose 15 mg: DE = 7.18; 95% CI: 2.85, 11.51). Reduction in PANSS symptoms appeared to mediate part of treatment effect (Paliperidone dose 3 mg: IE_panss_ = 3.03; 95% CI: 0.44, 5.62. Paliperidone dose 9 mg: IE_panss_ = 3.18; 95% CI: 0.78, 5.58. Paliperidone dose 15 mg: IE_panss_ = 3.78; 95% CI: 1.21, 6.36). The pathway through WG was not statistically significant, most likely owing to the low power and short follow-up of the study. However, we observed an interesting pattern in the indirect effect through WG. Although for the lowest dose of 3 mg the indirect effect through WG was positive, the direction of the effect was negative at higher doses (Paliperidone dose 3 mg: IE_wg_ = 0.28; 95% CI: − 2.29, 2.85. Paliperidone dose 9 mg: IE_wg_ = −0.18; 95% CI: −2.36, 1.99. Paliperidone dose 15 mg: IE_wg_ = −1.07; 95% CI: −4.85, 2.72). This result is driven by the significant negative quadratic association between WG and social functioning and the significant positive effect of paliperidone on WG. Although the treatment-mediator and mediator-outcome association were statistically significant, their magnitude was not large enough to yield a significant indirect effect in the mediation analysis. Meta-analyses of paliperidone trials and long-term follow-up studies are in progress to confirm this weak finding. By taking the ratio between the indirect effect through PANSS symptoms and the total effect (also called proportion mediated, PM) we estimated that part of the observed beneficial effect of paliperidone on PSP was owing to its effects on symptoms reduction (Paliperidone dose 3 mg: PM = 36%. Paliperidone dose 9 mg: PM = 33%. Paliperidone dose 15 mg: PM = 29%).

**Table 4:** Mediation analysis results

	Paliperidone 3 mg	Paliperidone 9 mg	Paliperidone 15 mg
DE	3.53 (− 0.40, 7.46)	5.16 (1.33, 8.99)	7.18 (2.85, 11.51)
IE_panss	3.03 (0.44, 5.62)	3.18 (0.78, 5.58)	3.78 (1.21, 6.36)
IE_wg	0.28 (− 2.29, 2.85)	− 0.18 (− 2.36, 1.99)	− 1.07 (− 4.85, 2.72)
TE	8.41 (4.37, 12.45)	9.62 (5.98, 13.26)	13.04 (9.12, 16.96)

All models adjusted for age, sex, race, country, relative day of disease onset, total PANSS score at baseline, PSP score at baseline, BARS score at baseline, SAS score at baseline, BMI at baseline

DE: direct effect; IE_panss: indirect effect through the sum of PANSS positive and negative (panss); IE_wg: indirect effect through percent weight gain (wg); TE: total effect

Estimates and CI are obtained via procedures developed in Bellavia and Valeri (2017). We report pooled estimates and pooled confidence intervals from the bootstrap analyses of 50 imputed data sets

## Discussion

The purpose of our study was to illustrate the use of approaches for mediation analysis in the presence of multiple mediators in the context of clinical trials for schizophrenia. Importantly, these approaches have wide applicability within the field of psychiatry trials and beyond. In particular, we aimed at investigating the interplay of psychiatric symptoms and adverse events, such as WG and extrapyramidal symptoms, in explaining the short-term effects of paliperidone treatment at different doses on patients’ social functioning. In this paper, we obtained clinically relevant findings along with insights on the methodological challenges in the application of mediation analyses approaches in this field.

By using recent developments in the field of causal interaction and mediation, we could quantify the role of PANSS, WG, and extrapyramidal symptoms as mediators of the treatment effect.^14^ We found that part of the clinically relevant improvement in social functioning achieved by paliperidone relative to placebo was due to reduction in PANSS symptoms, which, depending on the dose, explained between 29% and 36% of the treatment effect. Given the short follow-up of the study, we did not observe sizeable increases in weight, which displayed a quadratic association with social functioning. There are suggestions that the extent of WG is associated with improvement in psychopathology.^23,24^ Our findings are not in contrast with previous work. We report that modest WG is associated with improvement of functionality. However, for excessive percent WG (above 20%) the sign of this association reverts for patients assigned to moderate doses of paliperidone; the same change of direction in effects of excessive WG was observed for the low-dose and high-dose groups as well, at percent WG levels of 33% and 25%, respectively. The mediation analyses provide suggestive evidence that through increases in weight, the effect of treatment at high doses might be reduced. However, longer follow-up studies are needed to establish the role of WG. A sizeable part of the total effect was explained by pathways independent of symptoms and adverse events. Other important predictors of social functioning, which might be affected by treatment such as cognitive function should be considered in future studies. Our analysis represents an approach for jointly evaluating the interplay of antipsychotic treatments and side-effects in explaining efficacy outcomes. By using recently developed methods for causal mediation analysis we could investigate and formally test the contribution of multiple secondary outcomes in explaining short-term treatment effects on social functioning. In our analyses, we found evidence of non-linear effects. We did not find evidence of interactions between mediators and the outcome. However, the study was powered to detect large interaction effects of the same magnitude as the main effects. In the context of antipsychotic drugs, the reportedly complex operating mechanisms may likely be due to the presence of both interactive and mediating mechanisms. Effects estimated within the counterfactual approach to mediation analysis can be interpreted in causal terms. For example, the direct effects reported in this paper yielded what the effect of the drug would be had a hypothetical intervention fixed the mediators so that the antipsychotic had to operate through other pathways.^15^ The indirect effect through PANSS indicates the effect that the antipsychotic would have if it could only change PANSS scores, but could not change the other mediators.

Our analyses have some limitations. First, our intent-to-treat analysis could be biased in the presence of informative drop-out. We limited this source of bias using multiple imputation techniques for missing data. Future studies should assess the impact of treatment discontinuation and adherence on our results.^22,25,26^ The assessment of the role of mediators was hampered by the small sample size and short follow-up. In particular, it is important to evaluate sustained improvements in functionality. However, even in this short-term trial, paliperidone was shown to be effective in changing measures of social functioning. This is of importance as early effects of antipsychotics predict long-term success of the treatment. Future studies should investigate sustained improvements in functionality as well. Furthermore, we acknowledge that restricting the analysis to a two groups comparison in which an antipsychotic is compared with placebo (rather than the multiple-arm fixed dose study used here) would improve power. However, mechanisms that explain the effect of antipsychotics on social functioning might be dose dependent and motivated separate investigation by dose level. Another limitation of the study is the large variability of most of the estimates, which prevented several effects to reach statistical significance. Mediation and interaction analyses require greater sample sizes than conventional statistical models for detecting significant mediated effects.^27,28^ It is important for future studies to incorporate questions on mediation and interaction from the phase of study design. A meta-analysis of paliperidone trials to confirm such findings is currently work in progress. Finally, the causal interpretation of our analyses relies on strong assumption of no unmeasured confounding of the mediator-outcome relationship and that no confounders of the mediator-outcome relationship are affected by the treatment. In the current study, we were not able to account for medication history, which could lead to biased estimates. The findings have therefore to be interpreted with caution.

In conclusion, through this analysis we provide an application of approaches for mediation analysis with multiple mediators in the presence of non-linear effects in the context of clinical trials for schizophrenia. The regression analyses yielded evidence that excessive WG might negatively influence social functioning. The mediation analysis uncovered that the short-term effects of paliperidone for the treatment of schizophrenia in terms of social functioning are partly influenced by the success in reduction of PANSS positive and negative symptoms.

### Data availability

The data that support the findings of this study are open access and available from the Yale University Open Data Access (YODA) Project upon submission of a data request.

## Electronic supplementary material

Supplementary Files
