# Supplementary material for: The role of PANSS symptoms and adverse events in explaining the effects of paliperidone on social functioning: a causal mediation analysis approach
Source: NPJ Schizophr. 2018 Jun 27;4:13. doi: 10.1038/s41537-018-0054-8 (PMC6021395; doi:10.1038/s41537-018-0054-8)
Supplement: Supplementary file 1 — Supplementary Files [file 41537_2018_54_MOESM1_ESM.pdf]

Example R Code for Mediation Analysis with Multiple Mediators

```
# load libary
library(plyr)
library(dplyr)
library(mice)
# set wd
# read dataset

###### imputation
meth.spec<-
 c("pmm","pmm","pmm","pmm","pmm","pmm","pmm","pmm","pmm","pmm","pmm","","","","","","
 ")
imp.order<-
 c("panss_sum_w0","bars_w0","max_srs","pss_w0","weight_w0","bmi_w0","srs_w0","weight_
 w6","pss_w6","pystdy_w0","panss_sum_w5")
md.pattern(data)
input<- data
ini<-mice(input,maxit=0,meth=meth.spec,pri=FALSE)
pred<-ini$pred
# set order
vis <- ini$vis
vis <-vis[imp.order]
# impute data 10 times
M=50
impute<-mice(input,m=M,maxit=10,seed=500,pred=pred,vis=vis)


####################################################################################
 #######
completedData<-complete(impute,M)
completedData$pgain<-(completedData$weight_w6-
 completedData$weight_w0)/completedData$weight_w0
completedData$pgain2<-(completedData$pgain)*(completedData$pgain)
md.pattern(completedData)


# 4 mediators
# allow for mediators x treatment interaction
#M1=pgain at week 6
#M2=panss sum at week 5
#M4=max srs score


# bootstrap
nboot<-5000
te<-array(NA,c(nboot,50))
nde.int<-array(NA,c(nboot,50))
nie.int<-array(NA,c(nboot,50))
cde<-array(NA,c(nboot,50))
pnie_m1<-array(NA,c(nboot,50))
pnie_m2<-array(NA,c(nboot,50))
pnie_m4<-array(NA,c(nboot,50))
```

```
###### For loop iterate 10 imputed dataset
for (i in 1:M){
  df<-complete(baseline.impute,i)
  df$pgain<-(df$weight_w6-df$weight_w0)/df$weight_w0
  df$pgain2<-(df$pgain)*(df$pgain)
  cc<-as.matrix(c(0,0,0,0,0,0,0,0,0,0,0,mean(df$pystdy_w0)))
  yodadata<-df%>%filter(pystdy_w0<0)
  colnames(yodadata)

  #placebo vs paliperidone dose 1
  #placebo vs paliperidone dose 2
  #placebo vs paliperidone dose 3
  #placebo vs olanzapine
  #yodadata_palidose<-yodadata[which(yodadata$TRTGRPC==1|yodadata$TRTGRPC==2),]  #3
  #yodadata_palidose<-yodadata[which(yodadata$TRTGRPC==1|yodadata$TRTGRPC==3),]   #9
  yodadata_palidose<-yodadata[which(yodadata$TRTGRPC==1|yodadata$TRTGRPC==4),] #15

  for (j in 1:nboot){
    #obtain a bootstrap sample
    yodadata_palidose1_boot<-yodadata_palidose[sample(1:nrow(yodadata_palidose),
 replace = TRUE),]

    #outcome regression to estimate the total effect of trt on social functioning
    #TRT-Y
    reg.te.pss<-
 lm(pss_w6~as.factor(trtbin)+pss_w0+panss_sum_w0+bars_w0+srs_w0+bmi_w0
             +age+as.factor(sexbin)+as.factor(racebin)+as.factor(country)+pystdy_w0
                   , data=yodadata_palidose1_boot)


    #mediator regressions to estimate the effect of trt on potential mediators:
    #M1=pgain_w6
    #M1_sq=pgain_w6^2
    #M2=panss_sum_w5

    #TRT->M1
 reg.m1<-lm(pgain~as.factor(trtbin)+pss_w0+panss_sum_w0+bars_w0+srs_w0+bmi_w0
              +age+as.factor(sexbin)+as.factor(racebin)+as.factor(country)+pystdy_w0
               , data=yodadata_palidose1_boot)

    #TRT->M2
    reg.m2<-
 lm(panss_sum_w5~as.factor(trtbin)+pss_w0+panss_sum_w0+bars_w0+srs_w0+bmi_w0
             +age+as.factor(sexbin)+as.factor(racebin)+as.factor(country)+pystdy_w0
               , data=yodadata_palidose1_boot)


    #outcome regression to estimate the effect of trt on social functioning
 adjusting


    reg.adj.pss.int<-lm(pss_w6~as.factor(trtbin)+pgain+pgain2+panss_sum_w5 +
                    pss_w0+panss_sum_w0+bars_w0+srs_w0+bmi_w0+age+as.factor(sexbin)+
                          as.factor(racebin)+as.factor(country)+pystdy_w0
                         , data=yodadata_palidose1_boot)
```

```
    betac.m1<-as.matrix(reg.m1$coefficients[3:(length(reg.m1$coefficients))])
    betac.m2<-as.matrix(reg.m2$coefficients[3:(length(reg.m2$coefficients))])

    x10<-coef(reg.m1)[1]+t(cc)%*%betac.m1
    x20<-coef(reg.m2)[1]+t(cc)%*%betac.m2

    #Compute the effects of interest
    #Total effect:
    te[,i][j]<-coef(reg.te.pss)[2]
    #CDE
 cde[,i][j]<-coef(reg.adj.pss.int)[2]
    #PNIE: beta1*theta2 (coefficient of trt in mediator regression * linear
 coefficient of  mediator in outcome regression +  ((E(M|a,c)-E(M|a*,c))^2)*quadratic
 coefficient of  mediator in outcome regression
  pnie_m1[,i][j]<-coef(reg.m1)[2]*coef(reg.adj.pss.int)[3]+((x10+coef(reg.m1)[2])^2-
 (x10)^2)*coef(reg.adj.pss.int)[4]
    pnie_m2[,i][j]<-coef(reg.m2)[2]*coef(reg.adj.pss.int)[5]
    nde.int[,i][j]<-cde[,i][j
    nie.int[,i][j]<-pnie_m1[,i][j]+pnie_m2[,i][j]
    print(i)
    print(j)
  }

}
```
